# Effects of saline water on soil properties and red radish growth in saline soil as a function of co-applying wood chips biochar with chemical fertilizers

**DOI:** 10.1186/s12870-023-04397-3

**Published:** 2023-08-07

**Authors:** Abu El-Eyuoon Abu Zied Amin

**Affiliations:** Soils and Water Department, Faculty of Agriculture, Assiut University, P.O. Box: 71526, Assiut, Egypt

**Keywords:** Bulk density, Red radish, Saline soil, Soil available nitrogen, Water holding capacity

## Abstract

**Background:**

Currently, using unconventional water sources in agriculture has become necessary to face overpopulation worldwide. Therefore, a pot experiment was carried out to evaluate the effects of irrigation with saline water in the presence of co-applied wood chips biochar (WCB) with chemical fertilizers on physicochemical properties and nutrient availability as well as growth parameters, and yield of red radish (*Raphanus sativus* L.) grown in the saline sandy soil.

**Methods:**

The WCB was added to the saline sandy soil at levels of 0 (control), 2.5, and 5% w/w. Then, this soil was cultivated by red radish plants and irrigated with saline water (5 dS m^− 1^). This experiment was performed in a randomized complete block design with three replicates.

**Results:**

Compared with the control treatment, WCB treatments increased significantly soil water holding capacity by 34.8% and 73.2% for levels of 2.5 and 5%, respectively. Soil pH decreased significantly in all WCB treatments. The relative increase in the total available nitrogen over the control was 30.1 and 103.5% for 2.5 and 5% wood chips biochar, respectively. Compared to the control, applying WCB at 2.5% led to an increase in the fresh root weight of red radish plants by 142.7%, while 5% caused a decrease in the fresh root weight of red radish plants by 29.4%.

**Conclusion:**

Recently, WCB represents an interesting approach to the rehabilitation of saline soils and the management of using saline water sources. It is recommended that combined application of WCB at a level of 2.5% with chemical fertilizers in order to improve red radish growth and nutrient retention in the saline sandy soil which preserves the ecosystem as well as increases productivity leading to the reduction of costs.

## Highlights

Wood chips biochar applications into saline soil increased water holding capacity.

Incorporated wood chips biochar with chemical fertilizers enhanced the availability of nitrogen and potassium in saline soil.

Combining wood chips biochar at 2.5% with chemical fertilizers in saline soil improved yield parameters of red radish under saline water irrigation.

Wood chips biochar at 2.5% incorporated with chemical fertilizers represents a promising strategy in saline agriculture.

## Introduction

The significant decrease in the quantities of freshwater is attributed to great population growth and climate changes, so finding new water resources became necessary, as well as using non-conventional water, especially saline water, became one of the solutions to meet the challenges of freshwater resources’ scarcity [1, 2]. Globally, climate changes increased soil salinization, and freshwater scarcity has affected crop production negatively [3, 4]. In arid and semi-arid regions, the quality of freshwater resources became deficient. In addition, most of the countries in these regions suffer from water poverty [2, 5]. In several cultivated soils around the world, the water used for irrigation is saline [6], as well as non-conventional water resources such as treated wastewater, have also been used in agriculture on a large scale, which has poor quality [1, 7].

Radish (*Raphanus sativus* L.) plant is considered a storage root and moderately sensitive crop to the salinity of the soils [8]. The radish was cultivated in ancient Egypt (2700 − 2200 BC) and used as common food as well as for its seed oil [9]. Red radish is one of the most important root vegetable crops belonging to the *Brassicaceae* family and is cultivated annually in many countries around the world because of its benefits such as high nutritional, and medicinal value [10, 11]. Red radish plays a vital role in the cosmetics and food industry due to its content of natural colorants. It is also beneficial for health because of anthocyanin which can scavenge free radicals and antioxidant activity as well as anti-cancer, anti-diabetes, anti-inflammation, antiatherogenic, and cardioprotective properties [12].

Globally, about one billion ha of salt-affected soils can become available for cultivation after treating them chemically, physically, and biologically [13]. Most agricultural soils in Egypt depend on irrigation from the Nile River due to the lack of rainfall [14]. About 33% of the irrigated soils in Egypt suffer from salinity problems, as one of the reasons for forming salt-affected soils in Egypt is secondary salinization resulting from using poor water in the irrigation process accompanied by high evaporation rates [15]. Indeed, soil salinity represents a problem facing food security and environmental sustainability worldwide [16]. Moreover, climate change has a dangerous effect on soils as it increases the salinity in arable soils [17]. Agricultural production sustainability may be damaged by soil salinity and salt leaching in several irrigated soils [18].

Biochar is a stable carbon-rich solid that is produced through a pyrolysis process of plant and animal waste between 200 and 900°C under conditions with no or little oxygen to decompose the biomass [19, 20]. Recently, many studies found that the production and application of biochar is a promising strategy to mitigate climate change, enhance soil properties, and increase crop productivity in modern agriculture [19, 21–23]. Wood biochar is considered to be an appropriate soil amendment because it enhances the capacity of nutrient retention in the soil; it is also a favorable factor for the recovery of nutrients [24, 25]. Biochar applications to saline soil lead to enhancing plant growth due to several mechanisms such as decreased sodium (Na) uptake via adsorption on biochar surfaces, physical entrapment of salts in biochar pores, improvement of soil properties, declining oxidation stress, and improving microbial activities [26] as well as reducing osmotic stresses [27]. Until now, no studies have highlighted the incorporation of biochar and chemical fertilizers to improve some physicochemical properties and yield of red radish grown in saline sandy soil. The current study presents an important vision about wood chips biochar incorporation with chemical fertilizers which can be an alternative strategy to the rehabilitation and management of saline soils and red radish productivity under saline water irrigation. Therefore, the goals of this study are to examine the effects of irrigation by saline water with co-applying wood chips biochar and chemical fertilizers into saline sandy soil on (1) soil properties, (2) nutrient availability, as well as (3) growth and yield of red radish. This study hypothesized that the combination of biochar with chemical fertilizers will maximize the benefits of biochar by improving the physical and chemical properties of the soil as well as enhancing red radish growth in saline sandy soil under irrigation with saline water.

## Materials and methods

### Biochar production

Wood chips were collected from a local carpentry workshop at Assiut City, Egypt, and compressed in a metal container, then tightly closed. The cover of the container had some small holes to get rid of volatile substances and gases. Wood chips were pyrolyzed at 270 °C under oxygen-limited conditions in an electric furnace for 6 h. The properties of wood chips biochar are shown in Table 1.

**Table 1:** Some physical and chemical properties of the soil and wood chips biochar used in this study. Each value ± standard error (SE) is the mean of three replicates

Property		Value ± SE
Soil		
Sand %		86.95 ± 0.09
Silt %		6.45 ± 0.03
Clay %		6.60 ± 0.12
Texture		Loamy Sand
Bulk density (Mg m^− 3^)		1.59 ± 0.03
TOM %		0.49 ± 0.02
CaCO_3_ %		24.90 ± 0.03
pH_(1:2)_		7.68 ± 0.005
EC_(1:1)_ (dS m^−1^)		3.98 ± 0.005
Soluble sodium (mg kg^− 1^)		1597.34 ± 47.00
Available nitrogen (mg kg^− 1^)		101.01 ± 2.73
Available potassium (mg kg^− 1^)		314.06 ± 6.27
Wood chips biochar		
pH_(1:10)_		6.55 ± 0.04
EC_(1:10)_ (dS m^−1^)		0.26 + 0.01
Soluble potassium (mg kg^− 1^)		32.38 ± 1.26
Soluble sodium (mg kg^− 1^)		11.58 ± 1.09
DOC (mg kg^− 1^)		878.12 ± 20.31
TOC (g kg^− 1^)		259.99 ± 5.70
Available phosphorus (mg kg^− 1^)		18.80 ± 2.62
Available potassium (mg kg^− 1^)		42.07 ± 1.48
Total nitrogen (g kg^− 1^)		6.65 ± 0.20

TOM: Total organic matter; EC: Electrical conductivity; DOC, dissolved organic carbon; TOC, total organic carbon

### Pot experiment

An open pot experiment was conducted on soil taken from 0 to 20 cm depth at the Extension Farm (27°16′ N latitude, 31°34′ E longitude) of the Faculty of Agriculture, Assiut University, Wadi El-Assiuty, Assiut, Egypt. The soil under study was classified according to US soil taxonomy as Entisols; Typic Torripsamments. The soil samples were air-dried and crushed to pass through a 2-mm sieve before conducting the pot experiment. Some properties of this soil are given in Table 1. Each plastic pot (18 cm depth × 16 cm base diameter × 20.8 cm top diameter) was filled with five kg of this soil. This experiment consisted of three levels of wood chips biochar (0, 2.5, and 5% w/w). The experiment was performed in a randomized complete block design with three replicates. Ten red radish seeds were planted in each pot on November 12, 2020, irrigated with tap water (0.43 dS m^− 1^) until germination. After ten days of germination, four plants were thinned in each pot, and the pots were irrigated with saline water (5 dS m^− 1^). Saline water was prepared in the laboratory from sodium chloride and calcium chloride in a ratio of 2:1, respectively. Then, plants were thinned in each pot to three plants after 21 days from planting. Irrigation was done according to the red radish plant’s needs, so about 350 ml of saline water was added in each irrigation period for the pot. Each pot was fertilized by 328 mg nitrogen (N) (equivalent to 157.4 kg N ha^− 1^), 92 mg phosphorus (P) (equivalent to 44.2 kg P ha^− 1^), and 217 mg potassium (K) (equivalent to 104.2 kg K ha^− 1^); all fertilizers were added in solution form. The nitrogen and phosphate fertilizers were added in four doses, while potassium fertilizer was added in three doses. The total amount of saline water added to each pot is 4200 cm^− 3^ throughout the season. After 63 days from planting, the red radish was harvested on January 14, 2021. The following red radish parameters were recorded: fresh plant, fresh roots, fresh shoots, and diameter mean of roots per pot. The root and shoot of red radish were washed with distilled water and oven-dried at 70 °C until the weight was stable. Thence, the weight of the dry shoot and root was recorded. Soil samples in this study were taken from each pot after harvesting, air-drying, crushing, and keeping for chemical analysis. This open pot experiment was performed at the Department of Soils and Water, Faculty of Agriculture, Assiut University, Assiut, Egypt.

### Analyses of biochar and soil

The determination of the particle size distribution of the soil was carried out using the pipette method [28]. The content of calcium carbonate (CaCO_3_) in the soil before conducting the experiment was measured using the calcimeter method [29]. Bulk density was determined in the disturbed soil after harvesting by placing 50 g of soil in a graduated cylinder of known weight and then knocking on the cylinder as well as the volume was measured [30]. The water holding capacity (WHC) in the soil was estimated by saturating the soil samples with distilled water for 48 h, and then the soil was placed into a Buchner funnel, where the soil has been exposed to a vacuum filtration system until the free water was removed. The wet soil samples were weighed and then dried at 105–110 °C for 24 h. WHC was determined by the difference between the mass of the oven-dried and the wet sample. The percentage of WHC was calculated by using the following equations [31]:

$$\% WHC (W/W)=\frac{wet soil \left(g\right)-dry soil \left(g\right)}{dry soil \left(g\right)} \times 100$$

$$\% WHC (V/V)=\% WHC \times bulk density$$

The total organic matter of wood chips biochar and soil before conducting the experiment was estimated via the dichromate oxidation procedure [32]. Dissolved organic carbon (DOC) in wood chips biochar was extracted with 0.5 potassium sulfate [33]. The DOC in the extract of wood chips biochar was determined using the oxidation method with dichromate at 100 °C and subsequent back titration of the unreduced dichromate by ferrous sulfate [34]. Soil pH was measured in 1:2 of a soil-distilled water suspension by a glass electrode [35], while pH in the wood chips biochar was measured in suspension (1:10) according to Amin [20]. Electrical conductivity (EC) was measured in soil extract (1:1) using an electrical conductivity meter [35], while EC of wood chips biochar was measured in extract (1:10) according to Amin [20]. The soluble potassium and sodium in extracts of soil samples and wood chips biochar were analyzed by flame photometer [35]. Total available nitrogen was determined by using 10 g of the air-dried soil extracted with 50 mL 0.5 M K_2_SO_4_, which was shaken for 2 h and then filtered [33]. The total available nitrogen including ammonium (NH_4_^+^-N) and nitrate (NO_3_^−^-N) in soil extracts was determined by the Kjeldahl method in two steps: (1) determination of NH_4_^+^alone in the extracts and (2) determination of the NO_3_^−^ in the extracts through adding Devarda’s alloy to convert the entire NO_3_^−^ into NH_4_^+^ [36]. Available phosphorus in wood chips biochar was extracted by 0.5 M sodium bicarbonate with pH 8.5 [37] and phosphorus in biochar extract was measured by colorimetric analysis using chlorostannous phosphomolybdic acid method in the sulphuric acid system [35]. Available potassium (K) in the wood chips biochar and soil samples was extracted in the soil with 1 M ammonium acetate, pH 7, and then analyzed by a flame photometer [38]. The total nitrogen in wood chips biochar was estimated after the digestion by a mixture of H_2_SO_4_-H_2_O_2_ [39]. Then, nitrogen in the digest was determined by the micro-Kjeldahl method [35].

### Plant analysis

The total nitrogen, phosphorus, potassium, and sodium (Na) in the dried shoot and root of red radish plant samples were determined after the digestion by a mixture of H_2_SO_4_-H_2_O_2_ [39]. The total nitrogen in all digests was determined by the micro-Kjeldahl method and phosphorus was measured colorimetrically by the chlorostannous phosphomolybdic acid method in the sulphuric acid system [35], also, potassium and sodium were analyzed by flame photometry method. The plant nutrient uptake (mg pot^− 1^) was calculated as the product of the (root and shoot) biomass and the total nutrient concentration of plants (root and shoot) using the following equation [22]:

$$\begin{array}{l}{\rm{Nutrient}}uptake\\\left( {mgpo{t^{ - 1}}} \right) = \\\frac{{{\rm{Nutrientconcentration}}\left( {{\rm{mg}}k{g^{ - 1}}} \right){\rm{inplantpart}}\left( {{\rm{drymatter}}} \right) \times {\rm{drybiomass}}\left( {{\rm{g}}po{t^{ - 1}}} \right)}}{{1000}}\end{array}$$

### Statistical analysis

All data obtained were statistically analyzed using a one-way analysis of variance (ANOVA) by the MSTAT-C program which is created by the Crop and Soil Sciences Department, Michigan State University, United States. Significant differences among treatments were carried out by Tukey’s honestly significant difference test (Tukey’s HSD) at the 0.01 and 0.05 levels of probability (p).

## Results

### Some physical and chemical properties of saline sandy soil

**Fig. 1:**
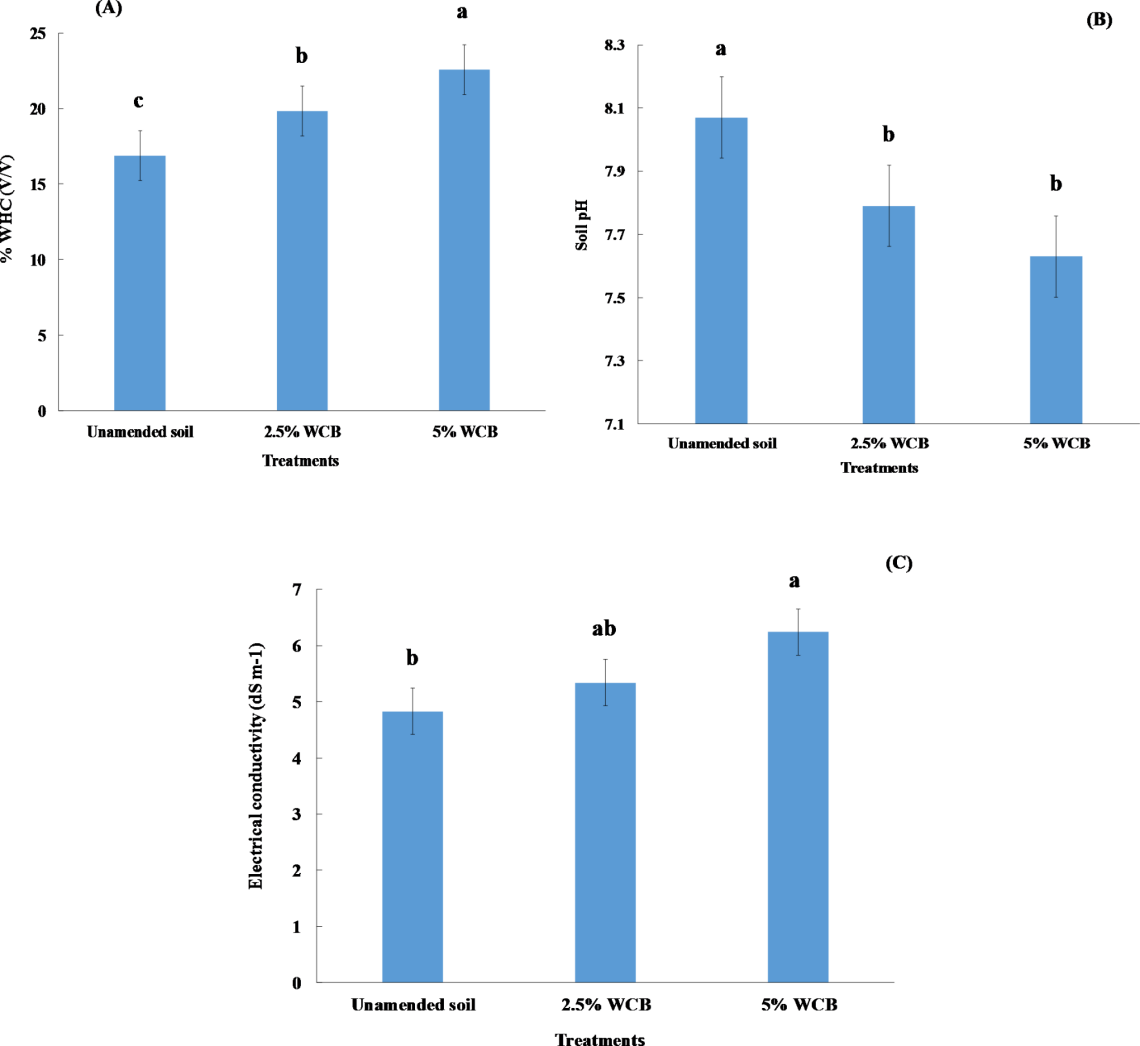
Effect of wood chips biochar (WCB) on water holding capacity (WHC), soil pH, and electrical conductivity (EC) in saline soil under irrigation with saline water. Each value represents the mean of three replicates with the standard error shown by the vertical bars. Different lowercase letters on each bar denote the significant differences among treatments according to Tukey’s Honestly Significant Difference test at p ≤ 0.05

Compared to the control treatment, wood chips biochar treatments decreased bulk density significantly (p ≤ 0.01) in saline sandy soil by increasing the amount of applied biochar. Bulk density values decreased from 1.58 Mg m^− 3^ for unamended soil to 1.38 and 1.22 Mg m^− 3^ for 2.5 and 5% wood chips biochar, respectively (Table 2). Water holding capacity increased significantly (p ≤ 0.0.05) with wood chips biochar applications at all doses after harvesting (Fig. 1A). The water holding capacity increased with increasing biochar doses. The values of water holding capacity increased from 16.87% (V/V) for unamended soil to 19.84 and 22.58% (V/V) for 2.5 and 5% wood chips biochar, respectively. Water holding capacity increased in 2.5 and 5% wood chips biochar treatments by 17.6% and 25.3%, respectively. Soil pH decreased significantly (p ≤ 0.05) in all wood chips biochar treatments after harvesting the red radish plants compared to the unamended soil (Fig. 1B). The soil pH decreased from 8.07 (unamended soil) to 7.79 and 7.63 for 2.5 and 5% wood chips biochar, respectively. The values of soil pH decreased with increasing biochar doses. Applying wood chips biochar decreased the soil pH by 0.28 and 0.44 units at doses of 2.5 and 5% amended soils respectively, relative to the unamended soil. In contrast, the electrical conductivity in the soil under study increased significantly (p ≤ 0.05) with applying wood chips biochar at a 5% dose compared to unamended soil (Fig. 1C). The electrical conductivity increased with increasing biochar doses. Electrical conductivity increased from 4.83 dS m^− 1^ (unamended soil) to 5.34 and 6.24 dS m^− 1^ for 2.5 and 5% wood chips biochar, respectively.

**Table 2:** Changes in bulk density and nutrient availability in saline soil after amending with wood chips biochar (All data are presented as the mean of three replicates ± standard error)

Treatment	Bulk density (Mg m^− 3^)	Nutrient availability (mg kg^− 1^ soil)			
		NH_4_^+^-N	NO_3_^−^-N	Total available nitrogen	Available potassium
Control	1.58 ± 0.02 a	41.25 ± 2.43 a	63.70 ± 1.05 c	104.95 ± 1.61 c	364.41 ± 3.42 c
2.5% WCB	1.38 ± 0.03 b	45.50 ± 1.05 a	91.00 ± 2.10 b	136.50 ± 2.78 b	433.52 ± 5.13 b
5% WCB	1.22 ± 0.02 c	46.11 ± 2.43 a	167.44 ± 0.00 a	213.55 ± 2.43 a	543.10 ± 11.40 a

Different lower case letters in each column denote the significant differences between treatments according to Tukey’s Honestly Significant Difference test at p ≤ 0.01

### Nutrient availability in saline sandy soil

After harvesting, the combined application of wood chips biochar and nitrogen fertilizers caused a significant increase (p ≤ 0.01) in total available nitrogen (NH_4_^+^-N + NO_3_^−^-N) in this soil compared to unamended soil (Table 2). The concentration of total available nitrogen increased with increasing wood chips biochar doses. Total available nitrogen increased from 104.95 mg kg^− 1^ soil for unamended soil to 136.50 and 213.55 mg kg^− 1^ soil for 2.5 and 5% wood chips biochar, respectively (Table 2). Adding wood chips biochar with nitrogen fertilizers increased NH_4_^+^-N insignificantly in this soil compared to unamended soil. The concentration of NH_4_^+^-N increased with increasing biochar doses. In comparison with unamended soil, wood chips biochar application with nitrogen fertilizers caused a significant increase of NO_3_^−^-N in the soil under study. The concentration of NO_3_^−^N increased with increasing biochar doses. The concentration of NO_3_^−^N increased from 63.70 mg kg^− 1^ soil for unamended soil to 91.00 and 167.44 mg kg^− 1^ soil for 2.5 and 5% wood chips biochar, respectively (Table 2). The application of wood chips biochar in the presence of potassium fertilizer caused a significant increase (p ≤ 0.01) in the soil’s available potassium compared to the unamended soil after harvesting. An increasing concentration of available potassium was observed with increasing wood chips biochar doses. After harvesting, available potassium increased from 364.41 mg kg^− 1^ soil for unamended soil to 433.52 and 543.10 mg kg^− 1^ soil for 2.5 and 5% wood chips biochar, respectively (Table 2).

### Parameters of red radish grown in saline sandy soil

**Fig. 2:**
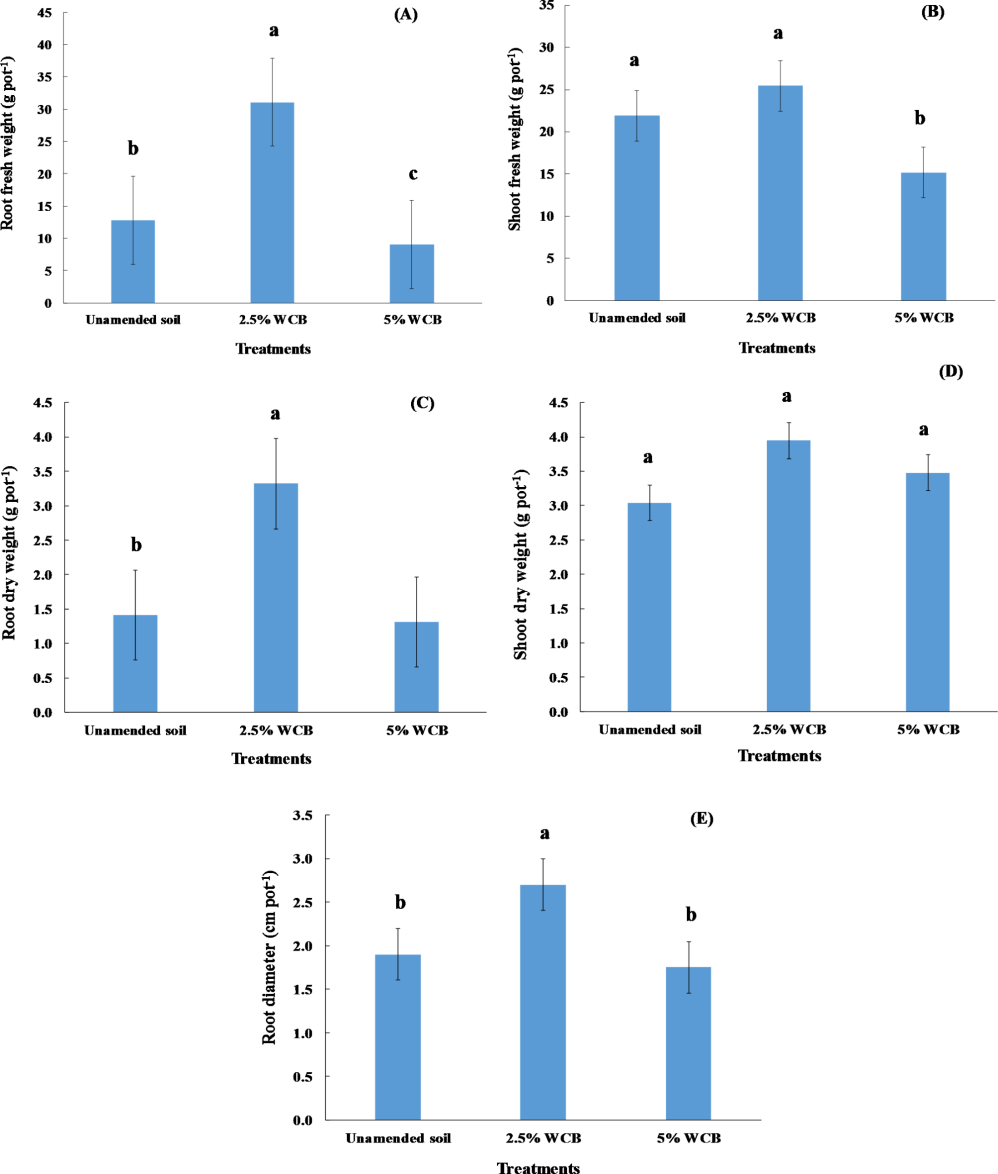
Effect of wood chips biochar (WCB) on the fresh and dry weight of root and shoot as well as root diameter of red radish plants grown in saline soil under irrigation with saline water. Each value represents the mean of three replicates with the standard error shown by the vertical bars. Different lowercase letters on each bar denote the significant differences among treatments according to Tukey’s Honestly Significant Difference test at p ≤ 0.05

Under irrigation with saline water, the application of wood chips biochar at a dose of 2.5% in saline soil increased significantly (p ≤ 0.05) the fresh root weight of red radish plants, but its application at a dose of 5% decreased significantly the fresh root weight of red radish plants compared to the unamended soil (Fig. 2A). Compared to control treatment, the wood chips biochar at dose 2.5% led to an increase in the fresh root weight of red radish plants by 142.7%, while its application at dose 5% caused a decrease in the fresh root weight of red radish plants by 29.4% (Fig. 2A). The fresh shoot weight of red radish plants was non-significantly influenced by the application of 2.5% wood chips biochar, but adding 5% wood chips biochar decreased significantly fresh shoot weight of red radish plants compared to the unamended soil (Fig. 2B). Moreover, the dry root weight of red radish plants increased significantly with applications of wood chips biochar at a dose of 2.5% compared to the control (Fig. 2C). The application of wood chips biochar at doses of 2.5 and 5% in saline soil increased non-significantly (p ≤ 0.05) the dry shoot weight of red radish plants compared to the unamended soil (Fig. 2D). Compared to the unamended soil, the root diameter of red radish plants increased significantly with applying wood chips biochar at a dose of 2.5%, but at a dose of 5% the root diameter of red radish plants decreased non-significantly (Fig. 2E). Applying wood chips biochar at doses of 2.5% led to an increase in the root diameter of red radish plants from 1.90 cm for unamended soil to 2.70 cm. Adding 2.5% wood chips biochar caused a root diameter increment of red radish plants by 42.1% (Fig. 2E).

### The concentration of nutrients in red radish grown in saline sandy soil

**Fig. 3:**
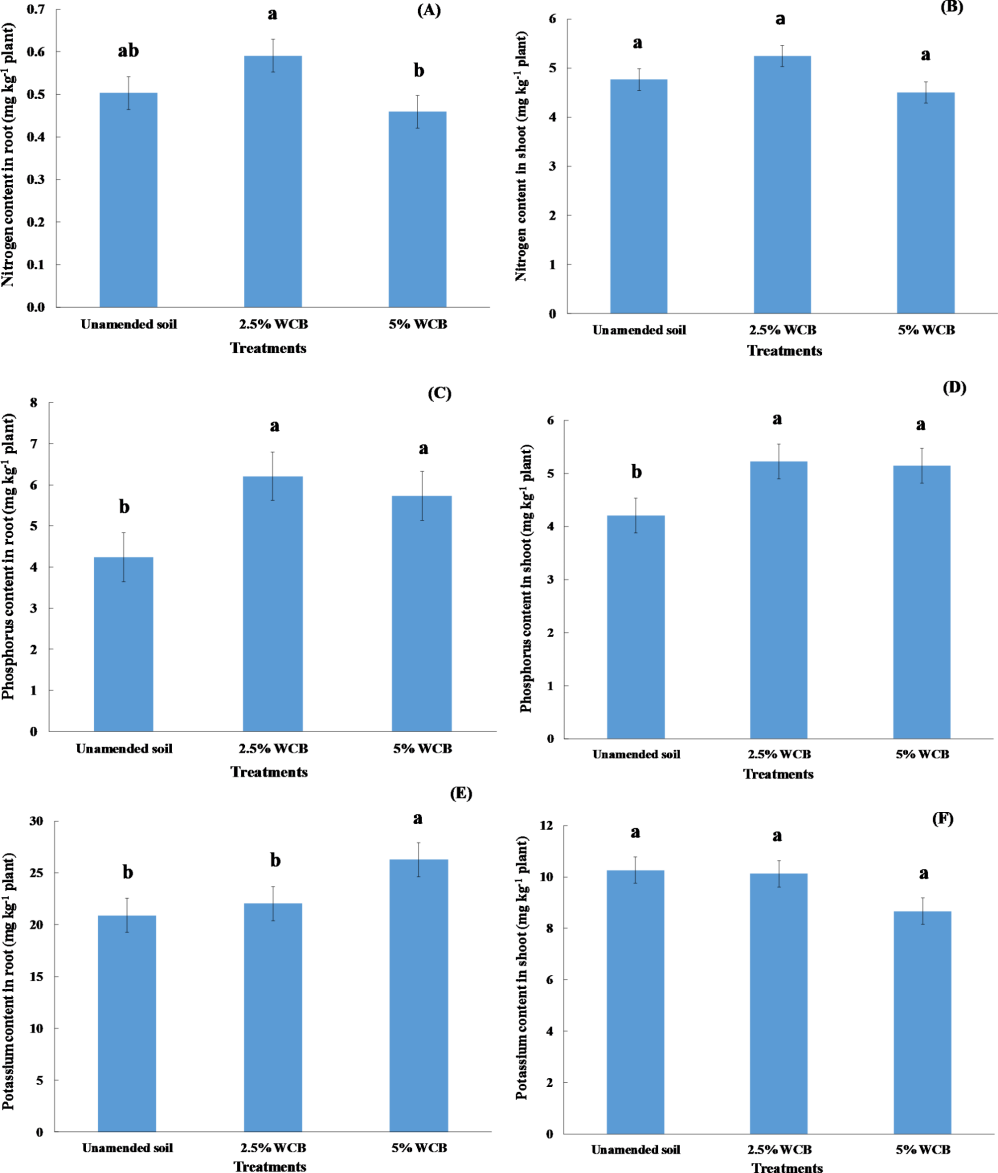
Effect of wood chips biochar (WCB) on nitrogen, phosphorus, and potassium contents in root and shoot of red radish plants grown in saline soil under irrigation with saline water. Each value represents the mean of three replicates with the standard error shown by the vertical bars. Different lowercase letters on each bar denote the significant differences among treatments according to Tukey’s Honestly Significant Difference test at p ≤ 0.05

**Fig. 4:**
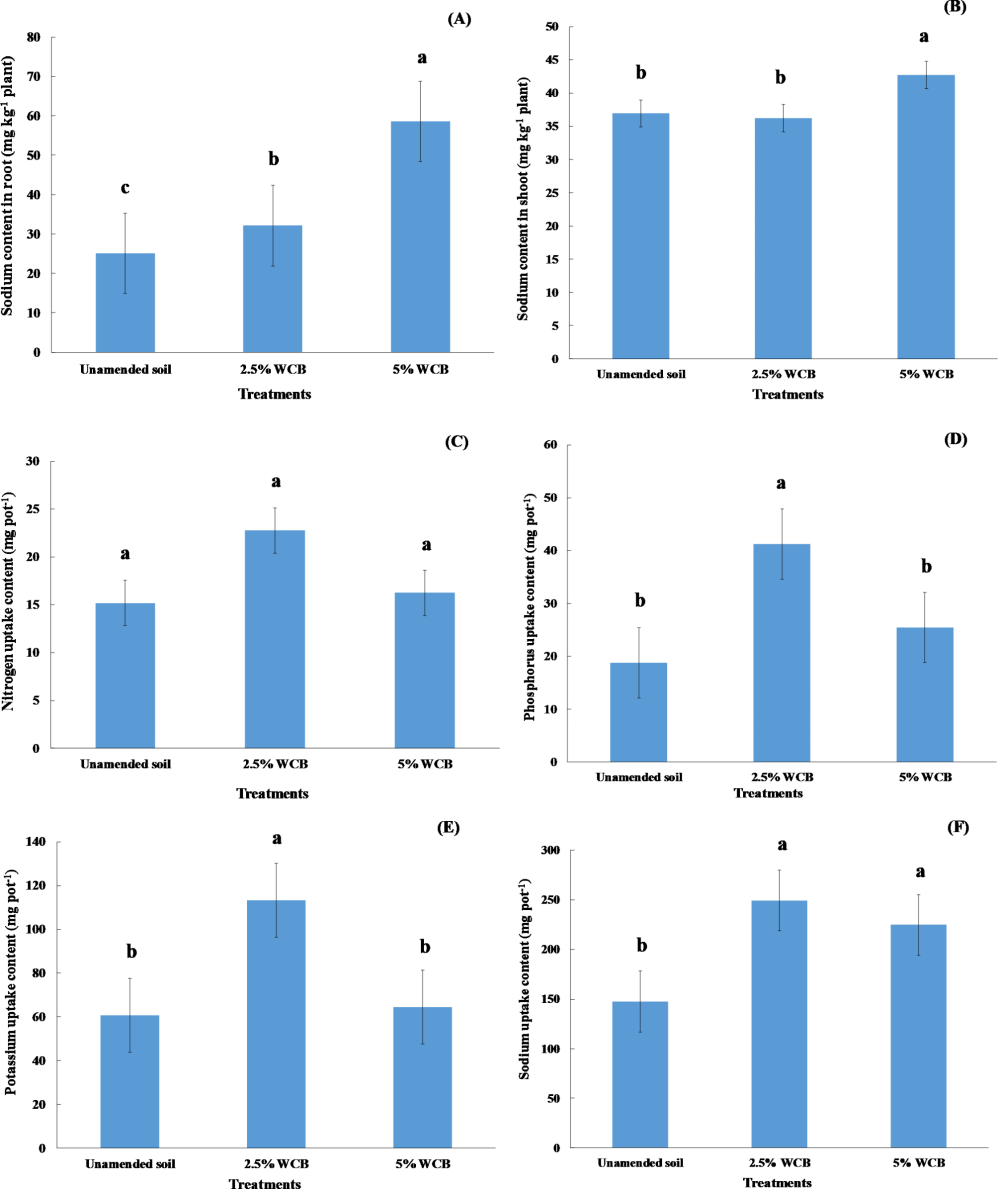
Influence of wood chips biochar (WCB) on sodium content in root and shoot as well as uptake content of nitrogen, phosphorus, potassium, and sodium by red radish plants grown in saline soil under irrigation with saline water. Each value represents the mean of three replicates with the standard error shown by the vertical bars. Different lowercase letters on each bar denote the significant differences among treatments according to Tukey’s Honestly Significant Difference test at p ≤ 0.05

The concentrations of nitrogen in roots and shoots increased non-significantly with applying 2.5% wood chips biochar compared with unamended soil, while they decreased non-significantly with adding 5% wood chips biochar (Fig. 3A and B). The applications of wood chips biochar at all doses into saline soil under irrigation with saline water led to a significant increase in concentrations of phosphorus in roots and shoots of red radish compared to the unamended soil (Fig. 3C and D). Wood chips biochar applications caused an increase in phosphorus in roots from 4.24 g kg^− 1^ (unamended soil) to 5.73 and 6.21 g kg^− 1^ plant for 5% and 2.5% doses, respectively (Fig. 3C), while its applications caused an increase of phosphorus in shoots from 4.20 g kg^− 1^ (unamended soil) to 5.14 and 5.22 g kg^− 1^ plant for 5% and 2.5% doses, respectively (Fig. 3D). The highest values of phosphorus in roots and shoots of red radish were observed at 2.5% wood chips biochar (Fig. 3C and D). Wood chips biochar application at 5% caused a significant increase of potassium concentration in the root of red radish compared to the control (Fig. 3E). Where wood chips biochar application in saline soil increased potassium concentration in root from 20.92 mg kg^− 1^ plant to 22.04 and 26.29 mg kg^− 1^ plant by 2.5 and 5%, respectively (Fig. 3E). However, there was no significant difference in potassium in the shoot of red radish among all treatments under study (Fig. 3F). A significant increase in the sodium concentration in the roots of red radish was noticed with applying wood chips biochar at doses of 2.5 and 5% compared to unamended soil (Fig. 4A). The sodium concentration in the root of red radish increased with increasing the doses of wood chips biochar (Fig. 4A). The sodium concentration in the shoot of red radish increased significantly with adding wood chips biochar at the dose of 5% (Fig. 4B).

### Nutrient uptake by red radish grown in saline sandy soil

The content of nitrogen uptake by red radish plants increased insignificantly with wood chips biochar additions to the saline soil. Nitrogen uptake content increased from 15.18 mg pot^− 1^ for unamended soil to 22.77, and 16.24 mg pot^− 1^ for 2.5% and 5% of wood chips biochar, respectively (Fig. 4C). Phosphorus uptake by red radish plants increased significantly with applying 2.5% wood chips biochar compared to the control (Fig. 4D). The content of phosphorus uptake increased from 18.73 mg pot^− 1^ for unamended soil to 41.20, and 25.45 mg pot^− 1^ for 2.5% and 5% of wood chips biochar, respectively (Fig. 4D). Potassium uptake by red radish plants increased significantly with adding 2.5% wood chips biochar compared to the unamended soil (Fig. 4E). The content of potassium uptake increased from 60.68 mg pot^− 1^ for unamended soil to 113.33, and 64.55 mg pot^− 1^ for 2.5 and 5% of wood chips biochar, respectively (Fig. 4E). The additions of wood chips biochar at doses of 2.5 and 5% led to a significant increase in sodium uptake by red radish plants compared to the unamended soil (Fig. 4F). Sodium uptake increased from 147.56 mg pot^− 1^ for unamended soil to 249.36, and 224.80 mg pot^− 1^ for 2.5% and 5% of wood chips biochar, respectively (Fig. 4F).

## Discussion

The effectiveness of biochar on soil properties depends on many factors such as experiment duration, soil type, and biochar levels [40]. The results obtained from this study (Table 2) were compatible with many researchers who found that the bulk density in sandy soil decreased with applying biochar and it also decreased with increasing biochar doses [41, 42] because of changes in structure, aggregation, and pore size of sandy soil [41]. Adding biochar to the sandy soil caused a reduction in its bulk density more than clay soil; this may be attributed to the variations in the size and density of biochar particles and sand compared to clay particles [43]. This result was in line with [41, 44, 45] who found that the incorporation of sandy soils with low-temperature biochar led to increasing water holding capacity, this may be a result of oxygen-containing functional groups’ presence on biochar surfaces, which would make biochar surfaces less hydrophobic [45], increase total porosity, aggregation, and structure of the soil, as well as provide a high internal surface area [41, 46]. Water holding capacity in sandy soil increased with increasing biochar doses [41] There are many factors controlling water holding capacity in the soil such as retention of water in intrapores, capillary water existing in intrapores, and the changes in interpores [24, 47]. biochar applications contribute to increases in the water holding capacity of sandy soil is attributed to the high porosity and large surface area of the biochar [31].

Wood chips biochar application decreased soil pH (Fig. 1B), this result agrees with Amin [48] who found that treating alkaline sandy soil with low-temperature biochar led to a decrease in the soil pH, because of the acidic functional group’s existence on its surfaces [49, 50]. Soil pH reduction can be explained in light of forming acidic compounds and releasing CO_2_ which is produced from chemical oxidation and biological decomposition of biochar in soil [45]. The application of biochar amendment to saline-alkali soil decreased pH values [51]. Moreover, increasing the doses of wood chips biochar produced at low temperatures caused the reduction of soil pH [48, 52]. Our results suggested that there was an increase in electrical conductivity with increasing levels of wood chips biochar (Fig. 1C). These results are compatible with Usman et al. [53] who reported that the electrical conductivity in sandy soil increased with increasing biochar doses under irrigation with saline water. The main reason for this increase may be attributed to the increased water holding capacity and its salts.

Applying biochar to the soils has many factors that play a role in nutrient retention such as type and doses of biochar as well as the properties and depth of the soils [54–56]. The nitrogen cycle in the soil is affected by wood biochar because of many processes including the adsorption of N, NO_3_^−^, NH_3_, and NH_4_^+^, in addition to the activity of soil organisms and microbial soil processes [24]. Our results were in agreement with Knoblauch et al. [57] who found that biochar-treated soils increased the content of available NO_3_^−^ and NH_4_^+^ significantly. Adding biochar improved the available NH_4_^+^ in saline soil [58]. In the present study, increasing available NO_3_^−^ and NH_4_^+^ with increasing biochar levels (Table 2). Similar results were reported by Wang et al. [51] who found the available NO_3_^−^ and NH_4_^+^ in saline-alkali soil increased with increasing biochar levels. Adding wood biochar combined with nitrogen fertilizer into the sandy soil decreased the concentrations of NO_3_^−^ and NH_4_^+^ leached, it also decreased with increasing wood biochar doses [59, 60]. Increasing retention of NH_4_^+^ in the soil occurred after applying biochar because of the increment of cation exchange capacity [61]. Biochar application with nitrogen fertilizers increased NO_3_^−^ retention in the soils this is attributed to improving water holding capacity [62, 63]. In the soils treated with biochar, the concentration of available NO_3_^−^ was higher than NH_4_^+^ concentrations because of increasing nitrification process activity [57]. Available potassium increased significantly with biochar application (Table 2) these results are similar to those of Naeem et al. [49] who reported that biochar-treated soils in the presence of potassium fertilizer resulted in an improvement in available potassium. In some of our previous studies, we found that adding wood chips biochar produced at low temperatures into the soils increased cation exchange capacity [48, 64]. This, in turn, leads to an increase of exchangeable potassium in the saline soil [64]. Amending the soil with biochar improves nutrient retention resulting in using fewer amounts of fertilizers and declining the climatic and environmental impact on the soils used for the cultivation of crops [65]. Incorporating biochar with nitrogen fertilizer can avoid N loss as well as enhance root growth and N uptake which in turn increases nitrogen utilization efficiency through enhancing soil properties such as pH, organic matter, and EC [66]. Promoting the use efficiency of fertilizers reduces nutrient loss in ecosystems as well as decreases the cost of adding fertilizers [67].

Our results present in (Fig. 2A, B, E) agree with the results from [68, 69] who showed that root weight, shoot weight, and root diameter of radish increased significantly with the application of biochar into the soil. The increase in root diameter resulting from biochar addition is attributed to the improvement of the physical properties of the soil [68]. In general, biochar additions increased nutrient content in red radish (Fig. 4A, B, C, D, and E). Our results are similar to those of Nabavinia et al. [69] who found that biochar applications to the soil caused a significant increase in concentrations of nitrogen and phosphorus in the root and shoot of radish plants, as well as Adekiya et al. [68] reported that adding biochar to the soil increased significantly the potassium content in the leaves of radish plants. Applying biochar to the sandy soil led to a significant increase in the soil organic matter, fruit yield, and nitrogen use efficiency of zucchini plants [22]. The increased yield parameters of radish plants were because of biochar’s ability to enhance nutrient availability in the soils [70, 71]. Biochar prepared at low temperature increased radish yield compared to biochar prepared at high temperature because it contains a high level of phosphorus availability [71]. Generally, the roots and leaves of radish plants are found to contain high concentrations of potassium [72]. Several studies found that amending soils with biochar caused an improvement in seed germination as well as the growth and crop yield of many plants [73, 74]. The amelioration of growth and productivity of plants in many sandy soils due to the additions of biochar is caused by many reasons: reducing bulk density as well as increasing total porosity and water holding capacity [75, 76], improving water use efficiency [77], it also enhances nutrient availability, organic matter, and cation exchange capacity [20, 22, 44], and improves soil biological properties [78]. In this study, it is obvious that applying biochar at 5% decreased the yield parameters of red radish plants in comparison with the rest of the treatments which may be attributed to the increased salt concentrations retained by the soil after using saline water in irrigation. Sanoubar et al. [79] found that exposing red radish to high concentrations of salt caused a decline in its root and shoot fresh weight. High salinity levels in the soil led to the reduction in fresh and dry weight of radish plants, which is a result of the specific effect of salts caused by the presence of toxic ions such as sodium (Na^+^) and chloride (Cl^−^), as well as hindering the plant’s absorption of water and nutrients [80]. According to the results of this study, the addition of biochar to the soils ensures the achievement that some of the United Nations’ sustainable development goals: clean water and sanitation, affordable and clean energy, responsible consumption and production, and climate action [81]. It is easy to apply this experiment in the field because there are no limitations as salt-affected soils and poor-quality water exist in the studied area. Nowadays, biochar is available in the local market. Saline agriculture is one such non-traditional solution that could revolutionize traditional agriculture, which contributes to solving the problems of water scarcity and increasing the demand for food. The mechanisms of management and rehabilitation of saline sandy soil under irrigation with saline water include (1) the addition of wood chips biochar at the level of 2.5%, (2) the cultivation of crops resistant or tolerant to salinity, and (3) mixing saline water with low salinity water during the irrigation process. These results confirm our hypothesis which assumes that adding wood chips biochar in saline sandy soil caused ameliorating physical and chemical properties, nutrient availability as well as improving red radish growth at adding 2.5% wood chips biochar. But, the hypothesis of improving red radish growth by adding 5% wood chips biochar was not confirmed.

## Conclusions

Globally, the increase in soil salinity represents a great challenge in agriculture as it accelerates soil degradation. In the current study, the doses of wood chips biochar played an important role in influencing the properties of saline sandy soil and yield parameters of red radish plants under irrigation with saline water. Wood chips biochar applications can be one of the most promising strategies used in saline sandy soil because of their great impact on some soil properties and fertility. Adding wood chips biochar into the studied soil led to enhancing water holding capacity, thus decreasing irrigation costs. The combined application of wood chips biochar and chemical fertilizers improved the availability of nitrogen and potassium compared to unamended soil. The wood chips biochar at dose 2.5% increased the fresh root weight of red radish by 142.7%, meanwhile, at dose 5% it reduced the fresh root weight of red radish plants by 29.4% in comparison with the control. Generally, wood chips biochar added at 2.5% decreased the effect of salinity produced from soil and irrigation water compared with a 5% level. Wood chips biochar applications are an innovative strategy for the rehabilitation and management of using saline soils and water, which is called saline agriculture in turn helps in the sustainable utilization of the available resources in the surrounding environment. From the results of this study, it is preferable to add wood chips biochar at a level of 2.5%. The practical importance of biochar application prevents nutrient loss which leads to protecting the ecosystem and reduces the cost of applying fertilizer.

## Data Availability

The datasets used or analyzed during the current study are available from the corresponding author upon reasonable request.
